# Identification of immune-related biomarkers and construction of regulatory network in patients with atherosclerosis

**DOI:** 10.1186/s12920-022-01397-4

**Published:** 2022-11-28

**Authors:** Ruoyu Dong, Guangwei Jiang, Yunjie Tian, Xiaoming Shi

**Affiliations:** 1 Department of Vascular Surgery, Hebei Provincial People’s Hospital, Shijiazhuang, 050000 China; 2 The Fourth Hospital of Hebei Medical University Gynecology, Shijiazhuang, 050000 China

**Keywords:** Atherosclerosis, RNA-Seq, Differentially expressed genes, Enrichment analysis, Immune

## Abstract

**Background:**

More and more evidence has established the crucial roles of the innate and adaptive immune systems in driving atherosclerosis-associated chronic inflammation in arterial blood vessels. Thus, the goal of this research was to determine immune-related biomarkers in atherosclerosis.

**Methods:**

In this study, we conducted analysis on the mRNA expression profile of atherosclerosis obtained from Gene Expression Omnibus. Differentially expressed genes (DEGs) between atherosclerosis and control samples and immune-related genes (IRGs) were intersected to obtain differentially expressed immune-related genes (DEIRGs). The protein–protein interaction (PPI) network was created by STRING database and hub genes were identified by the MCODE plug-in. Furthermore, the receiver operating characteristic (ROC) curve was executed to verify the diagnostic value of the hub genes, and microRNA (miRNA)-gene-transcription factor (TF) regulatory networks were used to explain the regulatory mechanism of hub genes in atherosclerosis. Finally, qRT-PCR was performed to identify the mRNA levels of the target genes.

**Results:**

A total of 199 overlapping genes were screened out as DEIRGs by intersecting the DEGs and IRGs. Then, 6 hub genes with high diagnostic value (IFIH1, IFIT1, IFIT2, IFIT3, ISG15 and OAS3) were identified via PPI network and ROC curve. Finally, miRNA-gene-TF networks revealed the regulatory mechanism of diagnostic genes.We used the carotid artery of AS patients and normal human carotid artery plaque samples for qRT-PCR verification, and the results showed that the hub gene had the same trend.

**Conclusion:**

Our study identified IFIH1, IFIT1, IFIT2, IFIT3, ISG15 and OAS3 as immune-related hub genes of atherosclerosis. These genes may serve as potential therapeutic targets for atherosclerosis patients.

## Introduction

Atherosclerosis(AS) is a type of arteriosclerosis that begins with homeostasis imbalance of vascular endothelial cells, accompanied by vasoconstriction and diastolic dysfunction  [1, 2]. Synthetic lesions such as mass metabolism disorders and release of inflammatory factors may cause a variety of disorders, such as stroke, coronary artery disease (CAD), peripheral artery disease (PAD), and other cerebrovascular diseases [3, 4]. Among them, stroke and coronary heart disease are considered to be the most common in the world [5]. Atherosclerotic is an important risk factor for these diseases. Therefore, it is crucial to treat and delay the atherosclerotic process [6]. AS is characterized by lipid buildup, narrowing the lumen of the arterial canal when atherosclerotic plaques form on the arterial wall [3]. The diagnosis of AS based on coronary angiography was regarder as gold standard, due to it is sexual examination and invasive [4, 7], clinical use is limited. Finding appropriate diagnostic markers is important for early detection of AS and and effective treatment of AS on time.

Previous studies have believed that atherosclerosis is caused by lipid deposition in the blood vessel wall, and as the study progresses [8]. It is gradually discovered that it is a chronic inflammatory disease of the arterial wall dominated by the aortic and middle arteries that is regulated by complex immune responses [9]. This chronic inflammatory response plays a key role in all stages of the atherosclerosis process, including atherosclerosis formation, progression, and plaque rupture eventually lead to thickening of the arterial wall lining, lumen narrowing, and even occlusion [10]. Compared with healthy individuals, patients with coronary heart disease have higher levels of CD8 + T cells in the blood. Human and mouse atherosclerotic plaques contain large numbers of CD8 + T cells. Depletion of CD8 + T cells using antibodies reduced the development of atherosclerosis in mice, suggesting that CD8 + T cells can promote atherosclerotic progression. so we suspect that immune-related genes play an important role in the development of AS [9, 11–13].

Our study used the AS transcriptome data in the GEO database, used the limma package to analyze the differential genes in AS. In order to further identify the role of immune-related genes in AS, we obtained the intersection with the immune-related genes from the Immport, TISIDB and InnateDB databases, and performed enrichment analysis, association analysis, etc. The hub gene was further screened by ROC curve and its diagnostic efficacy was evaluated.

## Materials and methods

### Data source

GEO is a free database of microarray/gene profile and next-generation sequencing. The expression profiles of human peripheral arteries from 92 atherosclerosis and 12 control tissues in the GSE100927 dataset were obtained as the training cohort. The expression profiles of 32 atheroma plaque samples and 32 control samples (distant macroscopically intact tissue) in the GSE43292 dataset were used for validation. We obtained 2,465 IRGs from Immport, TISIDB and InnateDB, which were comprehensive databases that curated IRGs from research articles, books, and digital resources.

### Identification of DEGs

DEGs between the atherosclerosis and control groups were identified using the “limma” *R* package. Statistically significant DEGs were defined with adj. *p*-value < 0.05 as the cut-off criterion. The analysis results were presented by volcano plot and heatmap.

### GO and KEGG enrichment analysis of DEIRGs

DEIRGs were obtained by intersecting these DEGs and IRGs. Gene ontology (GO) term analysis and Kyoto Encyclopedia of Genes and Genomes (KEGG) pathway analysis were performed using the R package “Org.Hs.eg.db” to identify the function of the DEIRGs [14]. GO terms or KEGG pathways with adj. *p*-value < 0.05 were considered statistically significant.

### Construction of PPI network and identification of hub genes

The PPI network of DEIRGs was constructed with the Search Tool for the Retrieval of Interacting Genes. The lowest interaction score should be greater than 0.4 and isolated nodes in the network were removed. Molecular Complex Detection, a Cytoscape plug-in, was used to identify the core module of the PPI network with default parameters as follows: degree cutoff = 2, node score cutoff = 0.2, and *k*-score = 2. And the genes in the core module were considered as the hub genes.

### ROC curve analysis of hub genes

The ROC curve, which was defined as a plot of test sensitivity as the y coordinate versus its 1-specificity or false positive rate (FPR) as the x coordinate, was an effective method of evaluating the quality or performance of diagnostic tests. The pROC package was employed to obtain the ROC curve and the value of the area under the ROC curve (AUC) in this work.

### Analysis of immune cell characteristics

The “Cell type Identification by Estimating Relative Subsets of RNA Transcripts (CIBERSORT)” algorithm was used to calculate the enrichment levels of 22 immune cell infiltration in atherosclerosis cohort. The difference in the immune cell infiltration between the atherosclerosis and normal groups was carried out using the Wilcoxon test. Moreover, we also analyzed the correlation between hub genes and differentially infiltrating immune cells.

### Functional similarity analysis and GSEA of hub genes

The functional similarity among proteins was evaluated using the geometric mean of semantic similarities in CCs and MFs through the “GOSemSim” *R* package. Moreover, to further explore the potential function of the selected hub genes in atherosclerosis, gene set enrichment analysis (GSEA) for single hub gene was performed. According to the median expression level of hub genes, sarcopenia samples were divided into two groups. The “c2.cp.kegg.v7.5.1.symbols.gmt” in Molecular Signatures Database (MSigDB) was selected as the reference gene set.

### Construction of regulatory network

The miRNet database, a network-based visual analysis tool was used to predict miRNAs and TFs that interact with the 6 hub genes. And we visualized the miRNA-gene-TF regulatory network by Cytoscape software.

### Patients and tissue samples

A total of 3 patients with pathologically confirmed AS in The Fourth Hospital of Hebei Medical University from Sep 2021 to Mar 2022 were collected in this study.

### Quantitative reverse

Transcription Polymerase Chain Reaction Total RNA was extracted from carotid artery tissues by RNA easylsolation Reagent (Vazyme #R701. Superscript IV ReverseTranscriptase(cat. no. 18,090,010, Thermo Fisher, Waltham, MA,USA)was performed for reverse transcription, and SYBRPremix Ex Taq II(Tli Rnaseh Plus)(cat. no. RR820QTakara, Kusatsu, Japan ) was utilized for the qRT-PCR in accordance with the instructions. The thermocycling program of qRT-PCR is shown below: initial denaturation for 30 s at 95C, 40 cycles of denaturation for 5 s at 95C, and 40 cycles of amplification for30 s at 60C. The mRNA expression of hub gene in cells and *β*-actin was used as the internal reference.

## Results

### Identification of DEGs in atherosclerosis

**Fig. 1:**
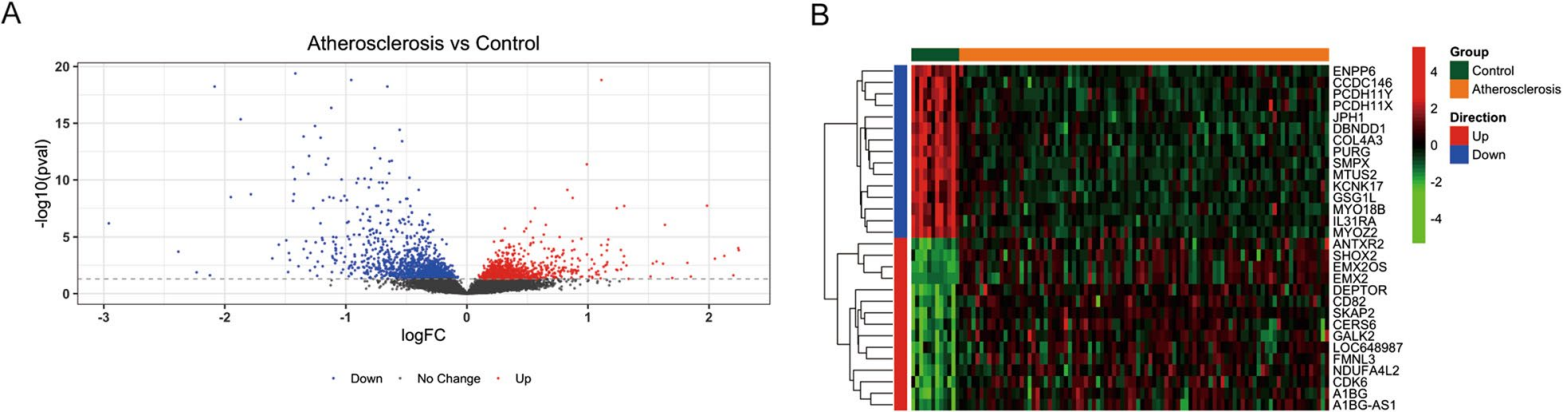
The differential expression genes identified by limma R package between AS patients and normal control. **A** Volcano plot for the differential expression analysis, blue for down-regulate gene, red for up-regulate. **B** Heatmap for the top 15 up-regulated genes and top 15 down-reguated genes as sorted by adjusted *p*-value

A total of 2,037 DEGs (atherosclerosis vs. control) were identified with the threshold at adj. *p*-value < 0.05, including 850 up-regulated genes and 1,187 down-regulated genes (Fig. 1 A). The expressions of the top 15 up-regulated genes and top 15 down-regulated genes (sorted by adj. *p*-value) were shown in Fig. 1B.

### Identification and enrichment analysis of DEIRGs

**Fig. 2:**
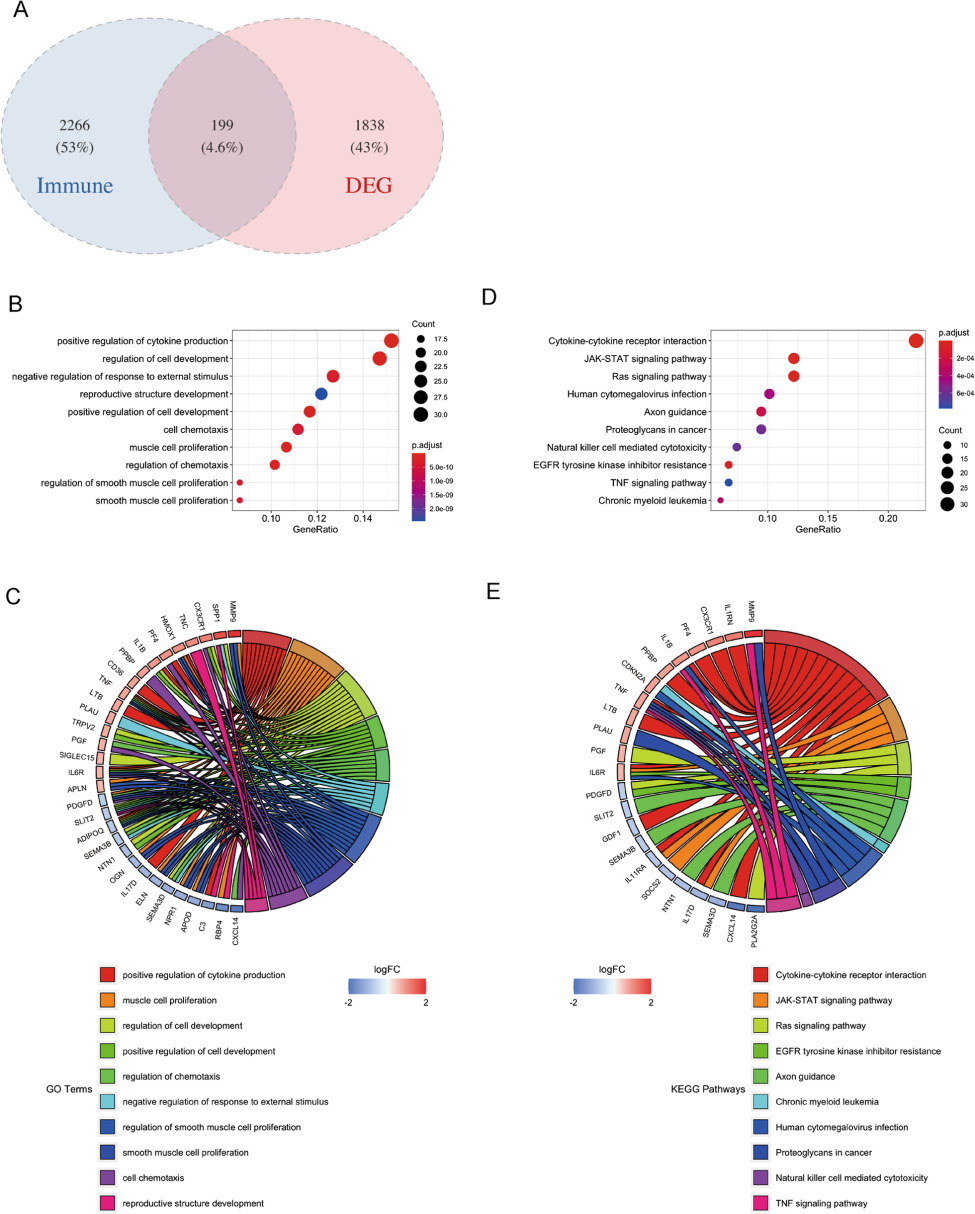
Differential genes associated with immunity. **A** The Venn diagram shows the intersection of DEG and immune-related genes. (**B**–**C**) GO enrichment results (**D**–**E**) KEGG enrichment results

199 overlapping genes from DEGs and IRGs were retained as DEIRGs for subsequent analysis (Fig. 2 A). Besides, the DEIRGs were enriched in different GO terms, such as “positive regulation of cytokine production”, “regulation of cell development”, “negative regulation of response to external stimulus”, “reproductive structure development” and “positive regulation of cell development” (Fig. 2B, C). The result of KEGG pathway enrichment [14] indicated that DEIRGs were mainly enriched in “cytokine-cytokine receptor interaction”, “JAK-STAT signaling pathway”, “Ras signaling pathway”, “human cytomegalovirus infection” and “axon guidance” and so on (Fig. 2D, E).

### Identification of hub genes by PPI network

**Fig. 3:**
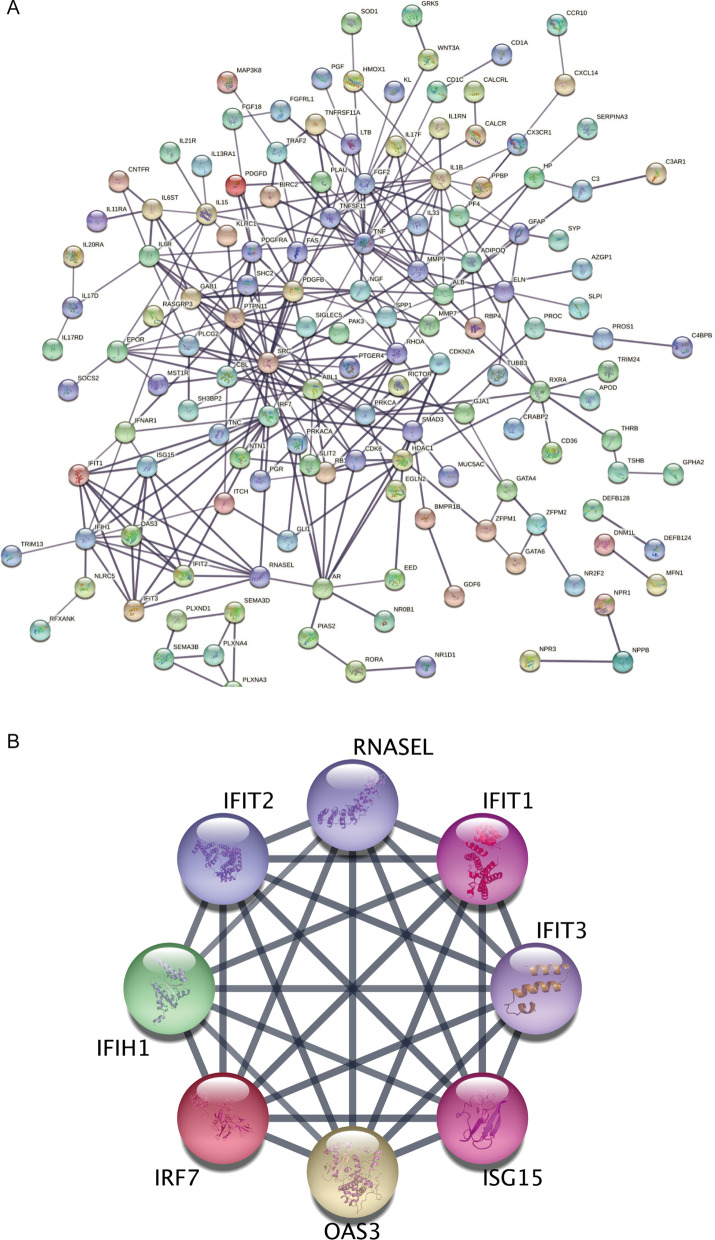
Protein network interaction and screening of key modules. **A** PPI protein network interaction diagram. **B** PPI network diagram of 8 hub genes from core module analyzed using MCODE.

To better understand the interplay among the identified DEIRGs, we used the STRING online server to construct a PPI network (Fig. 3A). Then, the core module was obtained from PPI network via the MCODE plug-in. There were 8 genes selected as hub genes, which were IFIH1, IFIT1, IFIT2, IFIT3, IRF7, ISG15, OAS3 and RNASEL (Fig. 3B).

### Validation of hub genes on IS

**Fig. 4:**
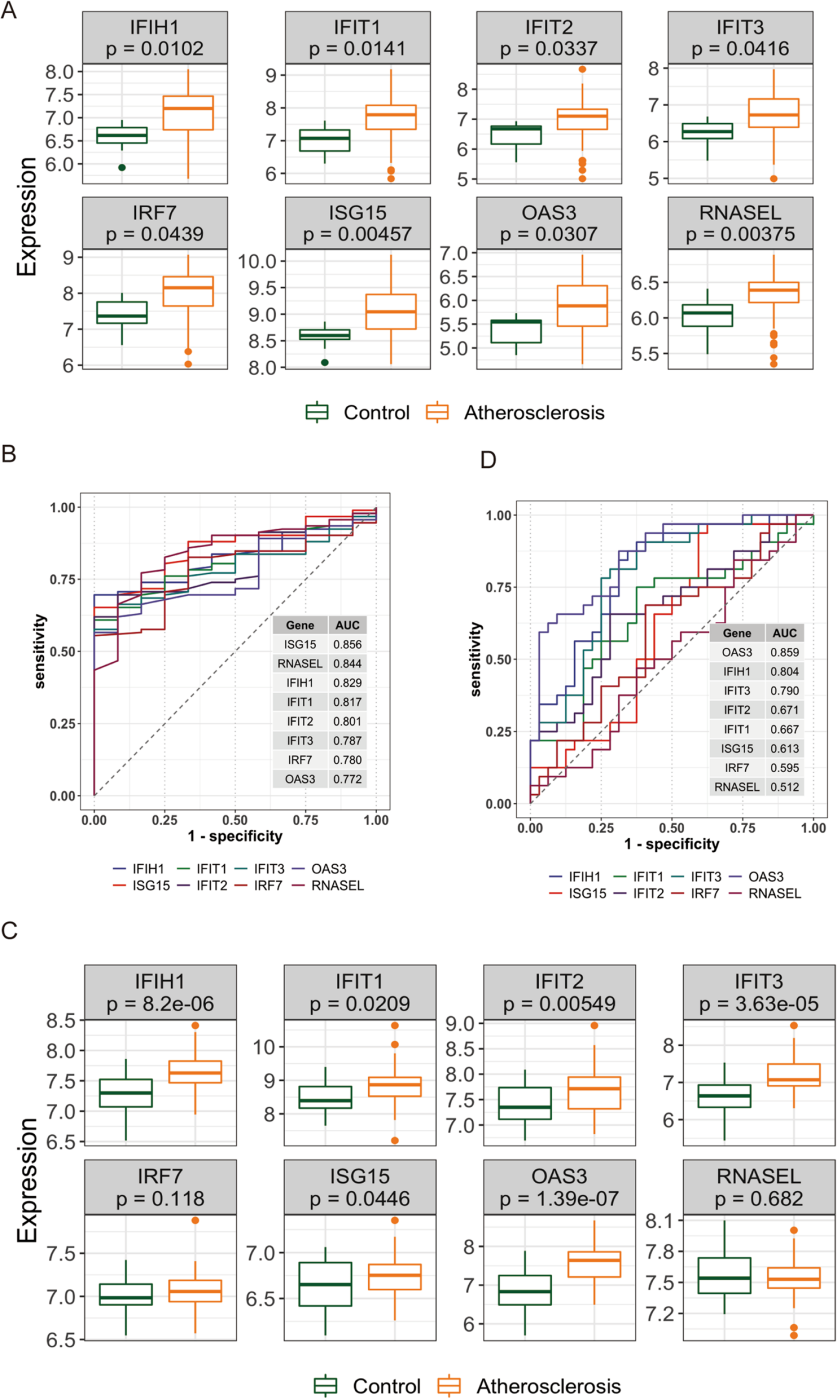
Evaluation of the diagnostic effect of hub gene on DKD. **A** Eight genes were expressed in both groups. **B** ROC curves and AUC values of the 8 hub genes. **C** Eight genes were expressed in both groups in valuation set. **D** ROC curves and AUC values of the 8 hub genes in valuation set

In GSE100927, IFIH1, IFIT1, IFIT2, IFIT3, IRF7, ISG15, OAS3 and RNASEL were all expressed higher in the atherosclerosis group compared to the control group (Fig. 4 A). Figure 4B showed that all the hub genes had good diagnostic ability. Next, the hub genes were further tested in the validation set GSE43292. IFIH1, IFIT1, IFIT2, IFIT3, ISG15 and OAS3 had similar expression differences to the training set (Fig. 4 C) and had good diagnostic abilities for atherosclerosis (Fig. 4D).

### Infiltrating immune cell analysis

**Fig. 5:**
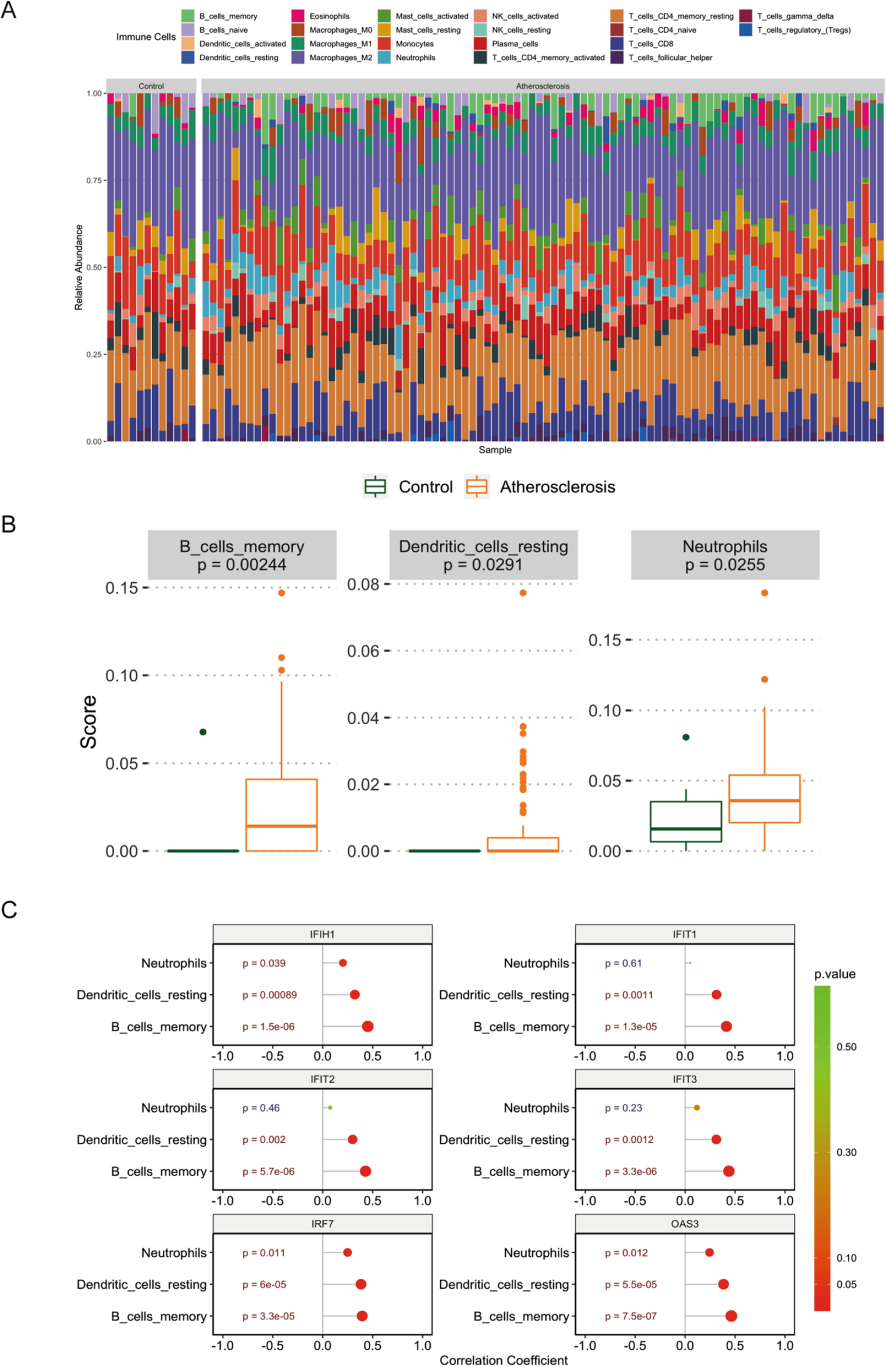
Comparison of immune infiltration between experimental groups in the control group. **A** 22 types of immune cells were found in both groups **B** Differential immune cells were compared between the two groups **C** Hub gene is associated with differential immune cells

Figure 5 A showed the distribution of 22 infiltrating immune cells in the atherosclerosis and normal samples. The result of the Wilcoxon test presented there were 3 types of immune cells with adjusted *P*-value < 0.05, which were memory B cells, resting dendritic cells and neutrophils (Fig. 5B). Moreover, the result of the correlations between hub genes and differentially infiltrating immune cells indicated that the memory B cells and resting dendritic cells were positively correlated with IFIH1, IFIT1, IFIT2, IFIT3, ISG15 and OAS3, and neutrophils were positively correlated with IFIH1 and OAS3 (Fig. 5 C).

### Functional similarity analysis and GSEA of hub genes

**Fig. 6:**
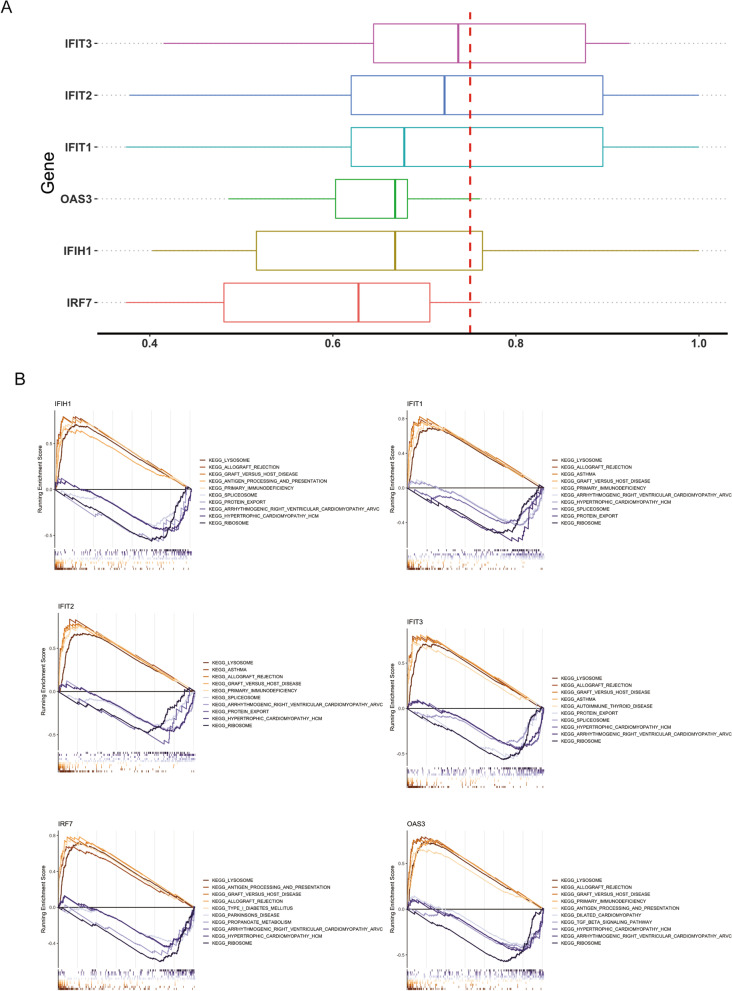
Hub gene correlation pathway analysis. **A** Hub gene semantic similarity analysis **B** GSEA analyzes the hub gene enrichment pathway

Moreover, we ranked the 6 hub genes based on the average functional similarity (Fig. 6 A). IFIT3, IFIT2 and IFIT1 were the top three proteins potentially playing key roles in atherosclerosis. Through GSEA of hub genes, we found that “KEGG_GRAFT_VERSUS_HOST_DISEASE”, “KEGG_LYSOSOME” and “KEGG_ALLOGRAFT_REJECTION” were enriched in highly expressed samples of all 6 hub genes, and “KEGG_RIBOSOME” was enriched in lowly expressed samples of all 6 hub genes (Fig. 6B).

### Construction of miRNA-gene-TF regulatory network

**Fig. 7:**
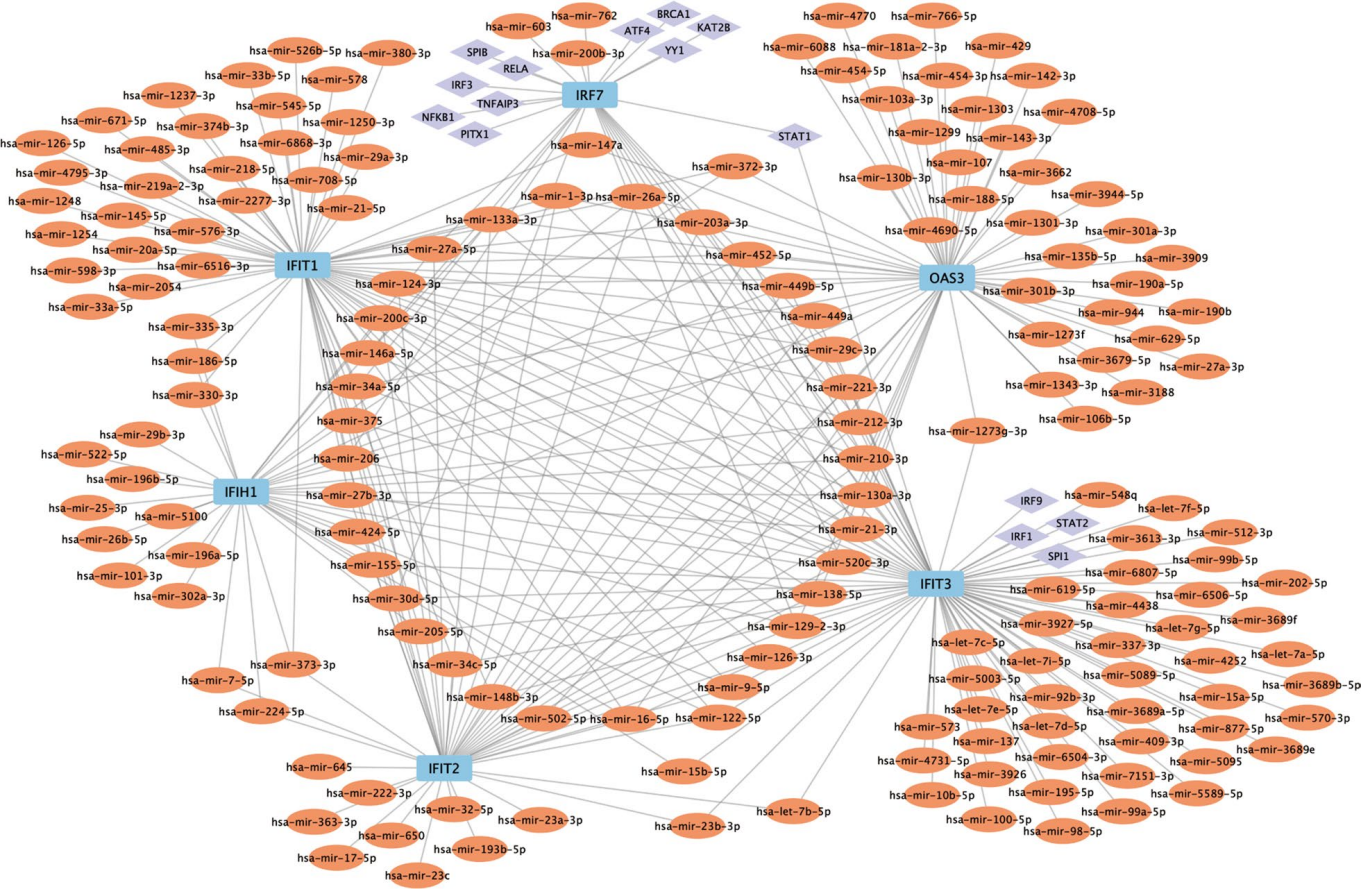
Construction of miRNA-gene-TF regulatory network. Light blue rectangle for hub genes, orange oval for miRNA, purple diamond for TF.

Subsequently, we predicted miRNAs and TFs of the 6 hub genes using the miRNet database. A total of 171 miRNAs and 36 TFs were identified that could potentially regulate the expressions of hub genes. Then, a miRNA-gene-TF regulatory network was constructed using Cytoscape to understand the possible regulation mechanism of hub genes (Fig. 7). hsa-mir-26a-5p, hsa-mir-212-3p, hsa-mir-449a, hsa-mir-129-2-3p, hsa-mir-21-3p, hsa-mir-130a-3p, hsa-mir-27a-5p, hsa-mir-133a-3p, hsa-mir-449b-5p, hsa-mir-210-3p and hsa-mir-16-5p could regulate all hub genes. And IFIT3 was the hub gene that was regulated by the largest number of miRNAs. For TFs, FOXC1 and GATA2 was the TF that regulated the largest number of hub genes. And IFIT1, IFIT3 and ISG15 were the top three hub genes regulated by the largest number of TFs.

### Verification of hub gene expression in carotid artery tissue samples

**Fig. 8:**
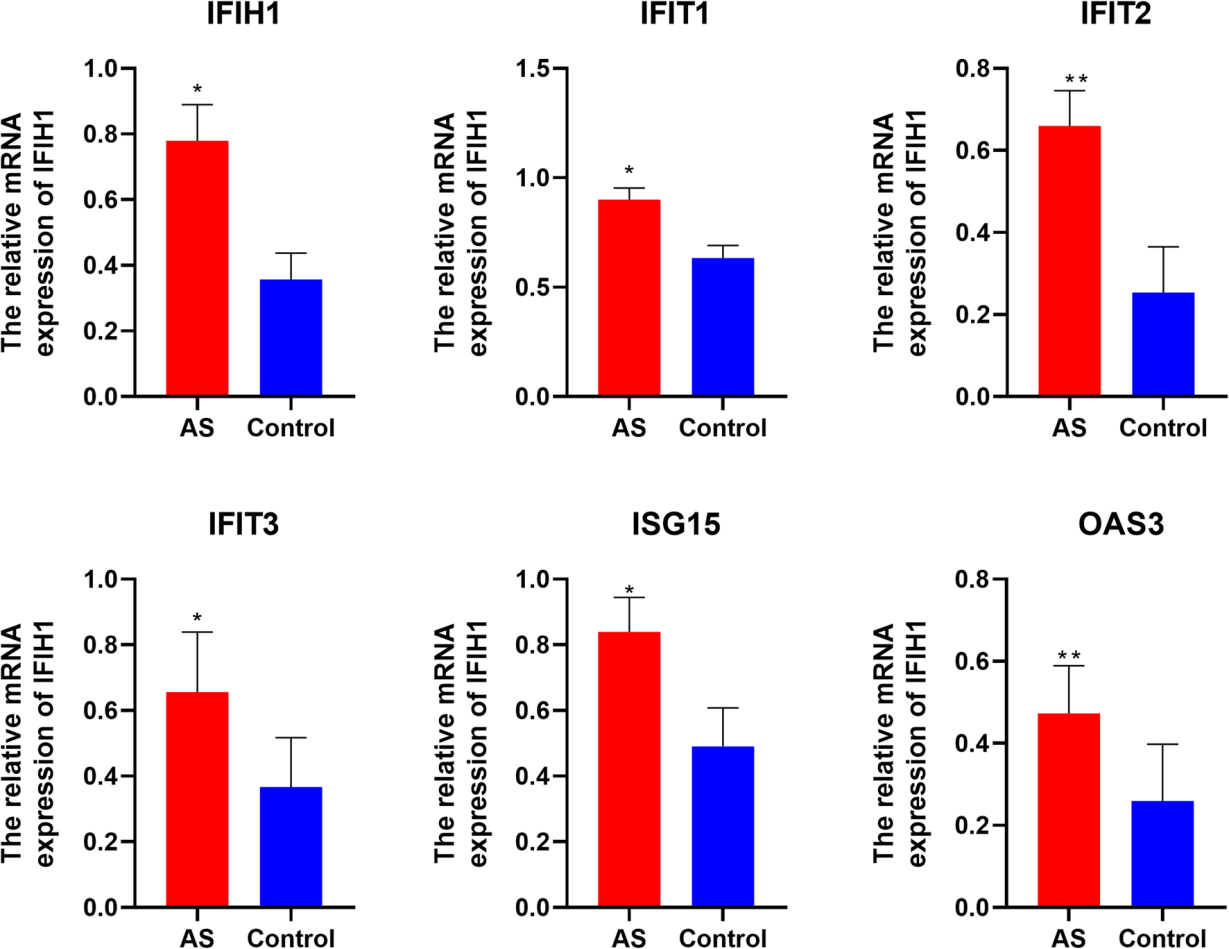
RT-qPCR validation of hub gene expression: MRNA expression of hub gene was evaluated in carotid artery patients’ tissue by qRT-PCR.

The mRNA expression of hub gene was evaluated in carotid artery patients’ tissue by qRT-PCR. As shown in Fig. 8, All hub genes were found to be upregulated in tissues of AS patients compared with the adjacent noncancerous tissues(*p* < 0.05), which is consistent with the results of the bioinformatics analysis.

## Discussion

There are many main etiological factors of atherosclerosis, including high-level cholesterol and low-density lipoprotein (LDL), low-level high-density lipoprotein (ihdl), hypertension, tobacco smoke, diabetes, obesity, unhealthy lifestyle, family history of heart disease, etc [2, 15]. However, the molecular mechanism of the AS has not been elucidated. With the development of molecular biology and cost reduction, gene sequencing analysis and precision therapy have become new diagnostic and therapeutic methods. In this study, R language was used to compare the differential genes of gse100927, and the immune related genes were selected. The results of enrichment analysis showed that the differential genes were mainly concentrated in the positive regulation of cytokine production, regulation of cell development and other pathways. Eight genes, which may be related to carotid atherosclerosis, were screened by constructing a protein interaction network. It was found that these genes have high diagnostic efficiency in as, and the network interaction map of TF and miRNA targeted by these genes was drawn.

In this study, gene expression data from newly diagnosed AS patients were analyzed using bioinformation approaches. At the gene expression level, we identify 177 DEIRGs that enriched in positive regulation of cytokine production, regulation of cell development, negative regulation of response to external stimulation and so on. Some pathways have been reported to play a role in AS. In Ren’s review, he pointed out that inflammation is a critical factor at all stages of atherosclerosis progression, proinflammatory cytokines accelerate atherosclerosis progression, while anti-inflammatory cytokines ameliorate the disease [16]. Abnormal cell development process also plays an important role in AS [17]. 8 hub genes (IFIH1, IFIT1, IFIT2, IFIT3, IRF7, ISG15, OAS3 and RNASEL) whose expressions were dysregulated in most patients and could potentially discriminate AS individuals. The memory B cells and resting dendritic cells were positively correlated with IFIH1, IFIT1, IFIT2, IFIT3, ISG15 and OAS3, and neutrophils were positively correlated with IFIH1 and OAS3. This suggests that these genes may regulate the development of AS by modulating the immune microenvironment of people with AS. IFIT1, IFIT2, IFIT3, IRF7, ISG15 are some of the hub genes that have been linked to the onset of AS. Interferon-induced protein with tetratricopeptide repeats 1 (IFIT1) mRNA is strongly upregulated in M1 polarized human primary macrophages, and besides IFITI is expressed in a subset of macrophages in aortic sinus and brachiocephalic artery from atherosclerotic ApoE-/- mice [18]. Zhang’s study suggested that IFN-induced ISG15, IFIT1-1, and IFIT1- 2 gene expression was increased in peripheral blood cells and aortic plaques, then lead to the formation of atherosclerotic plaque [18]. However, their study was limited to the possible role of individual genes in AS, while our study revealed the diagnostic performance of multiple genes including these genes in AS, and also conducted a study on the pathways in which these hub genes are located. Enrichment analysis, mining its potential pathways, etc.

Previous research has found that in AS individuals, islets are frequently associated by strong validation responses, indicating that immune system may play a role in the development of AS [19]. Correlation between hub genes expression and immune cell was analyzed to see if these hub genes may impact AS by affecting the immune system’s response. The results showed these hub genes were strongly indicated that the memory B cells and resting dendritic cells were positively correlated with IFIH1, IFIT1, IFIT2, IFIT3, ISG15 and OAS3, and neutrophils were positively correlated with IFIH1 and OAS3. The innate immune response dominated by monocytes and macrophages and the adaptive immune response caused by T cells, B cells, etc. constitute a complex immune network of atherosclerosis [20]. Clinical treatment of atherosclerotic cardiovascular disease for intrinsic immunity is relatively mature, as evidenced in the Shen’s study to reduce cardiovascular recurrence events by using IL-1β antibodies in patients with a history of heart disease who still have inflammatory residues after standard treatment [21].

Although the current study is the first to use GEO database analysis with a large sample size to analyze the metabolism related hub genes in AS, it does have limitations. On the one hand, we did not investigate the actual mechanism of the AS hub genes found. In contrast, we used data from only one GEO data set in our validation of hub gene diagnostic effectiveness. One more data set should be included to increase the credibility of our research. As a result, greater research into the expression of these hub genes in different races is required. The results of these studies all suggest that immunity plays an important role in AS, but our study reveals for the first time the correlation between these hub genes and immune cells in AS patients, which provides an important theoretical basis for a more accurate understanding of the role of immunity in AS.

In conclusion, according to our findings, the hub genes, and several functional biological pathways associated with immune response, metabolism and cytokines are all potential implicated in the etiology of AS. Although the actual molecular mechanism of hub genes and functional route in DM still must be investigated further, these findings bring fresh insights into the genesis of AS.

## Data Availability

The datasets for this study can be found in the GEO database. GSE100927, (https://www.ncbi.nlm.nih.gov/geo/query/acc.cgi?acc=GSE100927), GSE43292, https://www.ncbi.nlm.nih.gov/geo/query/acc.cgi?acc=GSE43292. The original sequenced samples in GEO are tissue type samples.

## References

[1] Nettersheim FS, Braumann S, Kobiyama K, Orecchioni M, Vassallo M, Miller J (2021). Autoimmune regulator (AIRE) deficiency does not affect atherosclerosis and CD4 T cell Immune tolerance to apolipoprotein B. Front Cardiovasc Med.

[2] Chen J, Zhang X, Millican R, Lynd T, Gangasani M, Malhotra S (2021). Recent progress in in vitro models for atherosclerosis studies. Front Cardiovasc Med.

[3] Surma S, Filipiak KJ (2022). Inflammation and autoimmunity in atherosclerosis. Reumatologia.

[4] Tang BY, Ge J, Wu Y, Wen J, Tang XH. The role of ADAM17 in inflammation-related atherosclerosis. J Cardiovasc Transl Res. 2022. 10.1007/s12265-022-10275-4 35648358

[5] Zhao L, Lv F, Zheng Y, Yan L, Cao X (2021). Characterization of an aging-based diagnostic gene signature and molecular subtypes with diverse immune infiltrations in atherosclerosis. Front Mol Biosci.

[6] Poels K, van Leent MMT, Boutros C, Tissot H, Roy S, Meerwaldt AE (2020). Immune checkpoint inhibitor therapy aggravates T cell-driven plaque inflammation in atherosclerosis. JACC CardioOncol.

[7] Vallejo J, Cochain C, Zernecke A, Ley K (2021). Heterogeneity of immune cells in human atherosclerosis revealed by scRNA-Seq. Cardiovasc Res.

[8] Liu H, Xiang C, Wang Z, Song Y (2022). Identification of potential ferroptosis-related biomarkers and immune infiltration in human coronary artery atherosclerosis. Int J Gen Med.

[9] Gao J, Shi L, Gu J, Zhang D, Wang W, Zhu X (2021). Difference of immune cell infiltration between stable and unstable carotid artery atherosclerosis. J Cell Mol Med.

[10] Kong P, Cui ZY, Huang XF, Zhang DD, Guo RJ, Han M (2022). Inflammation and atherosclerosis: signaling pathways and therapeutic intervention. Signal Transduct Target Ther.

[11] Alcaraz MJ, Alcaraz A, Teruel-Montoya R, Campillo JA, de la Torre A, Munoz A (2021). Subclinical atherosclerosis and immune activation in young HIV-infected patients with telomere shortening. Aging.

[12] Lee CF, Carley RE, Butler CA, Morrison AR (2021). Rac GTpase signaling in immune-mediated mechanisms of atherosclerosis. Cells..

[13] Li W, Bai X, Hao J, Xu X, Lin F, Jiang Q, et al. Thrombosis origin identification of cardioembolism and large artery atherosclerosis by distinct metabolites. J Neurointerv Surg. 2022;11(2):78–93. 10.1136/neurintsurg-2022-019047 35654581

[14] Ogata H, Goto S, Sato K, Fujibuchi W, Bono H, Kanehisa M (1999). KEGG: kyoto encyclopedia of genes and genomes. Nucleic Acids Res.

[15] Nawaz B, Fromm A, Oygarden H, Eide GE, Saeed S, Meijer R (2021). Prevalence of atherosclerosis and association with 5-year outcome: the norwegian stroke in the young study. Eur Stroke J.

[16] Ren Y, Qiao W, Fu D, Han Z, Liu W, Ye W (2017). Traditional Chinese Medicine protects against cytokine production as the potential immunosuppressive agents in atherosclerosis. J Immunol Res.

[17] Mahmoud AD, Ballantyne MD, Miscianinov V, Pinel K, Hung J, Scanlon JP (2019). The human-specific and smooth muscle cell-enriched LncRNA SMILR promotes proliferation by regulating Mitotic CENPF mRNA and drives cell-cycle progression which can be targeted to limit vascular remodeling. Circ Res.

[18] Huang C, Lewis C, Borg NA, Canals M, Diep H, Drummond GR (2018). Proteomic identification of interferon-induced proteins with tetratricopeptide repeats as markers of M1 macrophage polarization. J Proteome Res.

[19] Fernandes das Neves M, Batuca JR, Delgado Alves J (2021). The role of high-density lipoprotein in the regulation of the immune response: implications for atherosclerosis and autoimmunity. Immunology.

[20] Fernandez DM, Giannarelli C (2022). Immune cell profiling in atherosclerosis: role in research and precision medicine. Nat Rev Cardiol.

[21] Shen Y, Xu LR, Tang X, Lin CP, Yan D, Xue S (2021). Identification of potential therapeutic targets for atherosclerosis by analysing the gene signature related to different immune cells and immune regulators in atheromatous plaques. BMC Med Genomics.

